# Complementary Effects of Interleukin-15 and Alpha Interferon Induce Immunity in Hepatitis B Virus Transgenic Mice

**DOI:** 10.1128/JVI.01030-16

**Published:** 2016-09-12

**Authors:** Marianna Di Scala, Itziar Otano, Irene Gil-Fariña, Lucia Vanrell, Mirja Hommel, Cristina Olagüe, Africa Vales, Miguel Galarraga, Laura Guembe, Carlos Ortiz de Solorzano, Indrajit Ghosh, Mala K. Maini, Jesús Prieto, Gloria González-Aseguinolaza

**Affiliations:** a Gene Therapy and Regulation of Gene Expression Program, Center for Applied Medical Research (CIMA), Pamplona, Spain; b Instituto de Investigación Sanitaria de Navarra (IdisNA), Pamplona, Spain; c Division of Infection and Immunity, University College London, London, United Kingdom; d Imaging Unit and Cancer Imaging Laboratory, CIMA, Pamplona, Spain; e Department of Morphology, CIMA, Pamplona, Spain; f Centre for Sexual Health and HIV Research, University College London, London, United Kingdom; g University Clinic of Navarra, Pamplona, Spain; h CIBERehd, University of Navarra, Pamplona, Spain; University of Florida

## Abstract

In chronic hepatitis B (CHB), failure to control hepatitis B virus (HBV) is associated with T cell dysfunction. HBV transgenic mice mirror many features of the human disease, including T cell unresponsiveness, and thus represent an appropriate model in which to test novel therapeutic strategies. To date, the tolerant state of CD8^+^ T cells in these animals could be altered only by strong immunogens or by immunization with HBV antigen-pulsed dendritic cells; however, the effectors induced were unable to suppress viral gene expression or replication. Because of the known stimulatory properties of alpha interferon (IFN-α) and interleukin-15 (IL-15), this study explored the therapeutic potential of liver-directed gene transfer of these cytokines in a murine model of CHB using adeno-associated virus (AAV) delivery. This combination not only resulted in a reduction in the viral load in the liver and the induction of an antibody response but also gave rise to functional and specific CD8^+^ immunity. Furthermore, when splenic and intrahepatic lymphocytes from IFN-α- and IL-15-treated animals were transferred to new HBV carriers, partial antiviral immunity was achieved. In contrast to previous observations made using either cytokine alone, markedly attenuated PD-L1 induction in hepatic tissue was observed upon coadministration. An initial study with CHB patient samples also gave promising results. Hence, we demonstrated synergy between two stimulating cytokines, IL-15 and IFN-α, which, given together, constitute a potent approach to significantly enhance the CD8^+^ T cell response in a state of immune hyporesponsiveness. Such an approach may be useful for treating chronic viral infections and neoplastic conditions.

**IMPORTANCE** With 350 million people affected worldwide and 600,000 annual deaths due to HBV-induced liver cirrhosis and/or hepatocellular carcinoma, chronic hepatitis B (CHB) is a major health problem. However, current treatment options are costly and not very effective and/or need to be administered for life. The unprecedented efficacy of the strategy described in our paper may offer an alternative and is relevant for a broad spectrum of readers because of its clear translational importance to other chronic viral infections in which a hyporesponsive antigen-specific T cell repertoire prevents clearance of the pathogen.

## INTRODUCTION

Worldwide, 350 million people suffer from chronic hepatitis B (CHB), and approximately 600,000 people die annually because of hepatitis B virus (HBV)-induced liver cirrhosis and/or hepatocellular carcinoma (1). The host immune response to HBV antigens is a critical factor determining the outcome of infection. While patients with self-limited, acute HBV develop strong, multispecific T cell responses to viral antigens, these responses are weak and narrowly focused in chronic HBV carriers (2, 3). In these patients, HBV-specific CD4^+^ and CD8^+^ T cells display an exhausted phenotype characterized by failure to proliferate and failure to produce gamma interferon (IFN-γ), tumor necrosis factor alpha (TNF-α), and interleukin-2 (IL-2) after stimulation with viral antigens (4, 5).

A cytokine that has received much attention for the treatment of chronic hepatitis B and C infections is IFN-α. As a recombinant protein, it has been demonstrated to be effective in a proportion of patients (6, 7); however, patients with high viral loads and normal serum transaminase levels seem particularly resistant to IFN-α therapy (8). While IFN-α was shown to have a direct degrading effect on viral DNA (9) and to induce the expansion and activation of NK cells (10), it did not effectively support the expansion and/or survival of CD8^+^ T cells from patients with CHB (8). Interestingly, IFN-α can facilitate the response of CD8^+^ T cells to IL-15 stimulation by inducing the expression of IL-15 receptor subunit alpha (IL-15Rα) (11). Moreover, there is evidence indicating that long-lasting persistence of IFN-α-primed CD8^+^ T cells is favored by their enhanced responsiveness to IL-15 (12).

IL-15 is a powerful stimulatory cytokine that plays a key role in lymphocyte function and homeostasis. It is involved in various activation, proliferation, and differentiation processes of CD8^+^ T cells (13), NK cells (14), and CD4^+^ T cells (15, 16). IL-15 has been reported to be capable of rescuing tolerant CD8^+^ T cells for use in adoptive immunotherapy of established tumors (17), and in combination with retinoic acid, it abrogated tolerance to dietary antigens (18). Importantly, hepatic overexpression of IL-15 has recently been implicated in inducing an anti-HBV response, possibly by mediating IFN-β induction (19).

This study explored the therapeutic potential of liver-directed gene transfer of IFN-α and IL-15, alone or in combination, in a murine model of chronic HBV (20) by use of adeno-associated virus (AAV) delivery. Despite their limitations, HBV transgenic (HBVTg) mice are widely used for elucidating immune responses in CHB and evaluating therapeutic strategies for CHB (21). To date, it has been shown that strong immunogens or immunization with HBV antigen-pulsed dendritic cells was able to alter the tolerant state of CD8^+^ T cells in these animals; however, the effectors induced were unable to suppress viral gene expression or replication (22, 23). The results presented here show that combining IL-15 with IFN-α resulted in a functional and specific CD8^+^ response, which was reflected by a decrease in the level of HBV core antigen (HBcAg) in the liver, and could confer partial immunity upon splenocyte transfer to HBV-infected recipients.

## MATERIALS AND METHODS

### Animals and manipulations.

HBVTg mice were kindly provided by Francis V. Chisari (20). Mice were bred and maintained under pathogen-free conditions at the animal facility of the University of Navarra. For experiments, they were matched for age (6 to 10 weeks), sex (male), and levels of HBV DNA and HBV surface antigen (HBsAg) in serum. Age-matched C57BL/6 wild-type (WT) males were purchased from Harlan Laboratories (Barcelona, Spain). The experimental design was approved by the Ethical Committee for Animal Testing of the University of Navarra.

For all procedures, animals were anesthetized by intraperitoneal injection of a mixture of xylazine (Rompun 2%; Bayer) and ketamine (Imalgene 500; Merial) (1:9, vol/vol).

HBVTg mice were injected intravenously (i.v.) with AAV at a dose of 1.5 × 10^12^ viral genomes (vg)/kg, except for the control group, which received 3 × 10^12^ vg/kg.

Blood was collected by bleeding from the retro-orbital plexus, and serum samples were obtained by centrifugation of total blood.

For adoptive transfer experiments, C57BL/6 mice were injected with a recombinant AAV vector (5 × 10^12^ vg/kg) carrying the HBV 1.3 genome as described previously (28) in order to establish an alternative model of chronic HBV infection. Donor cells, consisting of 10^6^ intrahepatic lymphocytes (IHL) and 10^7^ splenocytes, were injected i.v. into the retro-orbital plexus in a final volume of 125 μl.

### Viral construction, production, and purification.

The recombinant AAV8 (rAAV8) vector with WT AAV2 inverted terminal repeats (ITRs) was produced as described previously (24, 25). The expression cassette in the AAV–IL-15 vector consists of the murine IL-15 gene (GenBank accession number DQ083237.1) under the regulation of a liver-specific promoter (25). The expression cassette in the AAV–IFN-α vector has been described previously and contains of the murine IFN-α1 gene under the regulation of the elongation factor 1α promoter (24). A luciferase-encoding AAV (AAV-LUC) served as a control (24).

### DNA and RNA analysis.

Total DNA and RNA were isolated from livers as described previously (25). Real-time PCR-based quantification of HBcAg and glyceraldehyde-3-phosphate dehydrogenase (GADPH) DNA and RNA was performed using SYBR green master mix (Applied Biosystems, Foster City, CA). The following primers were used: HBcAg sense (5′-TTCGCACTCCTCCAGCTTAT-3′) and antisense (5′-GGCGAGGGAGTTCTTCTTCTA-3′), GAPDH sense (5′-TGCACCACCAACTGCTTA-3′) and antisense (5′-CAGAAGACTGTGGATGGCCCCTC-3′), and luciferase sense (5′-TCGAGGAGCCTTCAGGATT-3′) and antisense (5′-TTTTGGCGAAGAAGGAGAAT-3′).

HBV DNA was isolated from 20-μl serum samples using the High Pure viral nucleic acid kit (Roche).

For Southern blot analysis of HBV replication intermediates, frozen liver and kidney tissues were mechanically pulverized, and total DNA was isolated and was digested with HindIII as described previously (20). Before electrophoresis, all DNA samples were digested with RNase A (Roche) at 10 mg/ml and 37°C overnight (o/n). Nylon filters (Hybond-N+; Amersham) were hybridized with a ^32^P-radiolabeled HBV-specific DNA probe.

### Determination of serum ALT levels.

Levels of alanine aminotransferase (ALT) in blood were measured using commercial kits (Sigma Chemicals, St. Louis, MO) and a Hitachi automatic analyzer (Boehringer Mannheim, Indianapolis, IN).

### ELISAs for IL-15, IFN-α, and HBsAg levels in serum.

The concentration of IL-15 in serum was determined using the Mouse IL-15/IL-15R Complex ELISA (enzyme-linked immunosorbent assay) Ready-SET-Go! set (eBioscience), and the serum IFN-α concentration was determined using the VeriKine Mouse Interferon Alpha ELISA kit (PBL Assay Science). HBsAg levels were also determined by ELISA (Bioelisa HBsAg 3.0; Biokit).

### Liver histology, IHC, and TUNEL staining.

Liver sections were fixed in 4% paraformaldehyde (Panreac), embedded in paraffin, sectioned (thickness, 5 μm), and stained with hematoxylin and eosin (H&E). Immunohistochemistry (IHC) for HBcAg (B0586; Dako, Glostrup, Denmark) and PD-L1 (ab58810; Abcam) was performed using the EnVision system (Dako) according to the manufacturer's recommendations. *In situ* detection of cells with DNA double-strand breaks was performed by the terminal deoxynucleotidyltransferase-mediated dUTP-biotin nick end labeling (TUNEL) staining method by use of an *In Situ* Cell Death Detection kit (Roche) according to instructions. Images were acquired digitally with an Axio Imager. MI microscope (Zeiss, Germany) using an in-house MetaMorph macro (Molecular Devices, USA). All images were stored in uncompressed 24-bit color TIFF format. Images were automatically analyzed using a plugin developed for Fiji, ImageJ, at NIH.

### Isolation of IHL.

Mice were perfused via the portal vein with 10 ml of Ca^2+^- and Mg^2+^-free phosphate buffer solution preheated to 37°C. After perfusion, the vena cava was cut and the liver extracted. After incubation in 10 ml of phosphate-buffered saline (PBS) containing 1,000 U of type II collagenase (Gibco) for 20 min, the liver homogenate was passed through a 70-μm nylon cell strainer, and the cell suspension was centrifuged at 1,200 rpm for 10 min. The cell pellet was resuspended in 40% Percoll and was centrifuged at 1,800 rpm for 10 min. Red blood cells were removed using a lysis buffer (Gibco); the remaining cells were washed and were resuspended in RPMI 1640 medium.

### *In vitro* analysis of lymphocyte proliferation.

Intrahepatic lymphocytes obtained from HBV transgenic mice treated with LUC, IFN-α, IL-15, or IL-15 plus IFN-α were isolated, labeled with carboxyfluorescein succinimidyl ester (CFSE), and cultured for 3 days in the presence or absence of 10 μg/ml of HBcAg peptide (epitope C93-100). The proliferation of CD8^+^ T cells was analyzed by flow cytometry.

### Antibody and pentamer staining.

Cells were labeled with the following antibodies according to the manufacturer's recommendations: allophycocyanin (APC)-conjugated CD19 (CD19-APC) (clone PeCa1; ImmunoTools), CD4-APC/Cy7 (clone GK1.5), CD44-APC/Cy7 (clone IM7), CD8a-Pacific Blue (clone 53.6.7), NK1.1-APC (clone PK136), PD-1 conjugated with fluorescein isothiocyanate (FITC) or brilliant violet 421 (clone 29F.1A12) (all from BioLegend), and CD8a-FITC (clone 1D3; eBioscience).

For pentamer staining. 2 × 10^6^ isolated cells were incubated for 10 min at room temperature (RT) with major histocompatibility complex (MHC) class I peptide pentamers (H-2Kb/ILSPFLPLL for HBsAg [epitope S_208–216_], H-2Kb/MGLKFRQL for HBcAg [epitope C_93–100_], or H-2Kb/SIINFEKL for ovalbumin [OVA] [epitope OVA_257–264_]; ProImmune). Cells were then washed and were stained with the respective surface antibodies according to the manufacturer's recommendations.

Data were acquired with an 8-color BD FACSCanto II system equipped with three lasers (488 nm, 633 nm, and 405 nm) or with a 4-color FACSCalibur system equipped with two lasers (argon [488 nm] and diode [635 nm]). Fluorescence-activated cell sorter (FACS) data were analyzed with FlowJo or DIVA software.

### *In vivo* killing assay (IVK).

Splenocytes isolated from HBVTg or WT mice were pulsed with HBV peptide C_93–100_ or S_208–216_, or with an irrelevant peptide (ovalbumin; epitope OVA_257–264_) (all from Think Peptides), for 30 min at 37°C. Cells were then labeled with 5 μM (for HBV peptides) or 0.5 μM (for the control peptide) CFSE (Invitrogen), and 5 × 10^6^ cells of each population were transferred intravenously to mice. Twenty-four hours later, animals were sacrificed, liver and spleen lymphocytes isolated, and CFSE staining analyzed by FACS.

### Depletion of lymphocyte subpopulations and PD-L1 blockade.

CD8 T cell depletion studies were performed by intraperitoneal administration of 100 μg of an anti-mouse CD8 antibody (clone H35-17.2). Doses were scheduled starting 3 days before vector injection and at days 0, 3, 7, and 14 after virus injection. Depletion levels were determined to be >98%. Irrelevant mouse immunoglobulin isotypes were used as controls. NK cell depletion studies were performed by intraperitoneal administration of 200 μg of anti-mouse NK1.1 antibodies (clone PK136). Doses were scheduled daily starting 2 days before virus injection and at days 1, 3, 7, and 14 after virus injection. Depletion levels were determined to be >98%. Irrelevant mouse immunoglobulin isotypes were used as controls.

For *in vivo* PD1–PD-L1 blockade, rat anti–mouse PDL-1 (clone 10F.9G2; catalog no. BE0101; BioXCell) or a rat IgG2b isotype-matched control antibody (LTF-2; catalog no. BE0090; BioXCell) was injected intraperitoneally 1 day before AAV–IL-15 or AAV-LUC injection and at days 0, 3, 6, and 9 after injection.

### Patients and isolation of peripheral blood mononuclear cells (PBMCs).

PBMCs were isolated by Ficoll gradient centrifugation from heparinized blood samples taken from a total of 21 patients with CHB. Ethical approval was obtained through UK NHS REC 11/LO/0421, and each patient gave written informed consent. All patients were negative for anti-HCV and anti-HIV antibodies and were treatment naïve. Patients had a median age of 40 years (range, 27 to 60 years), and 2/21 were positive for HBV envelope antigen (HBeAg). The median HBV DNA level was 1,400 international units (IU)/ml (range, below the limit of quantification [BLQ] to 2 × 10^8^ IU/ml), and the median ALT level was 31 IU/liter (range, 18 to 474 IU/liter).

### HBV peptide stimulation of human PBMCs.

Patient PBMCs were stimulated with 1 μM overlapping long peptides spanning the whole HBV core protein (genotype D [ayw]; JPT Peptide Technologies) in the presence or absence of IFN-α (10^3^ IU/ml), IL-15 (10 ng/ml; R&D), or both. Cells were cultured in complete RPMI medium supplemented with 20 IU/ml IL-2 for 10 days at 37°C. IL-2 and medium were refreshed on day 4 of culture. On day 9, PBMCs were restimulated with 1 μM HBV peptide in the presence of brefeldin A (1 μg/ml; Sigma) for 16 h. For intracellular cytokine staining, cells were fixed and permeabilized using a Cytofix/Cytoperm kit (BD Biosciences) for 15 min at 4°C and were then stained with phycoerythrin (PE)-conjugated anti-granzyme B (anti-GrB; Biolegend) and anti-IFN-γ BV421 (BD) for 30 min at 4°C. Samples were analyzed using a FACS LSRII flow cytometer (BD Biosciences).

### Statistical analysis.

Data are presented as means ± standard errors of the means (SEM) and were analyzed for significance by the Student *t* test. Differences among groups were analyzed by one-way analysis of variance (ANOVA) followed by a Bonferroni multiple-comparison test using GraphPad Prism software, version 5.0. (Significance is indicated by asterisks in the figures as follows: *, *P* < 0.05; **, *P* < 0.01; ***, *P* < 0.001; ****, *P* < 0.0001.)

For analysis of the reduction of viral replication and the decrease in the number of HBV core-positive nuclei induced by AAV–IFN-α combined with AAV–IL-15, the fold inhibition (FI) for each treatment was calculated as the ratio of the level of viral RNA obtained from the livers of mice treated with AAV-LUC to the level of viral RNA obtained from the livers of mice treated with AAV–IFN-α (FI_IFN-α_), AAV-IL-15 (FI_IL-15_), or both (FI_combination_). The synergy index (*S*) for the combination of AAV–IFN-α and AAV–IL-15 was calculated as described previously (47) from the fold inhibition by use of the equation *S* = FI_combination_ − (FI_IFN-α_ + FI_IL-15_)/(FI_IFN-α_ + FI_IL-15_). The critical value of *S* is zero and indicates an additive effect, while a positive value for *S* shows synergism.

## RESULTS

### Antiviral effect of IL-15–IFN-α combination treatment in HBVTg mice.

**FIG 1:**
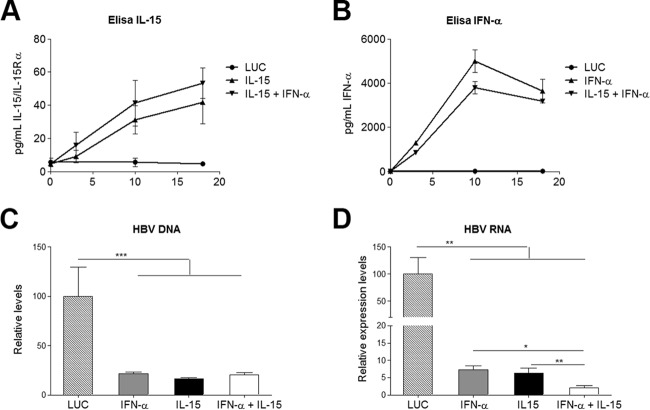
Cytokine expression and reduced HBV nucleic acids. (A and B) Concentrations of the IL-15–IL-15Rα complex (A) and IFN-α (B) in serum after vector injection. Results are representative of 3 independent experiments and represent means ± SEM for triplicates. (C and D) Ten days after vector injection, animals were sacrificed and livers assessed for the presence of HBV DNA (C) and HBV RNA (D). Data were normalized to GADPH levels, and the means ± SEM for 5 to 6 animals/group are shown. *, *P* < 0.05; **, *P* < 0.01; ***, *P* < 0.001.

The immunomodulatory potential of IL-15 and IFN-α in a chronic HBV setting was investigated using an HBVTg mouse model (20). HBVTg mice and C57BL/6 WT mice received a single intravenous (i.v.) injection of AAV–IFN-α, AAV–IL-15, a combination of both, or an equivalent dose of a control vector expressing the luciferase reporter gene (LUC). Serum cytokine levels were measured at different time points after vector injection (Fig. 1A and B).

**FIG 2:**
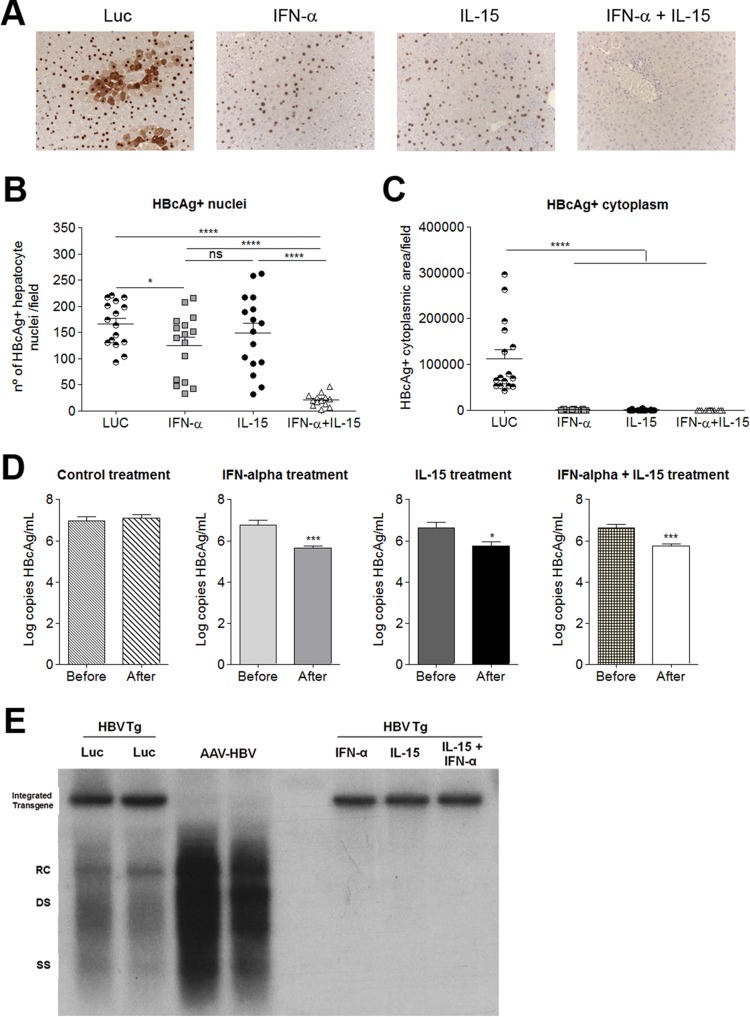
Synergistic antiviral effect of combined IL-15 and IFN-α treatment. (A) HBcAg protein expression in the liver, determined by IHC. A representative image is shown for each treatment group. (B and C) Fields were analyzed automatically to determine the number of hepatocytes positive for HBcAg in the nucleus (B) or the area of HBcAg staining in the cytoplasm (C). Each symbol represents the mean value for 10 different sections per mouse. Means ± SEM for each group (*n* = 16) are also shown. (D) HBV genome copy numbers 10 days after vector injection. Graphs show means (*n* = 5) ± SEM. *, *P* < 0.05; ***, *P* < 0.001; ****, *P* < 0.0001; ns, nonsignificant. (E) HBV replicative intermediates in the liver by Southern blot analysis 10 days after vector injection. RC, relaxed-circular DNA; DS, double-stranded DNA; SS, single-stranded DNA; Luc, HBVTg mice treated with AAV-Luc; AAV-HBV, C57BL/6 WT mice receiving AAV-HBV; IFN-α, HBVTg mouse treated with AAV-IFN-α; IL-15, HBVTg mouse treated with AAV-IL15; IFN-α + IL-15, HBVTg mouse treated with AAV-IFN-α + AAV-IL-15. Results from representative animals from each treatment group are shown.

To evaluate the direct antiviral effect elicited by the different treatments, total HBV-specific DNA and RNA in the liver were analyzed by quantitative real-time PCR. IFN-α, IL-15, and the combination treatment exerted similar inhibitory effects on HBV replication (Fig. 1C). However, transcriptional inhibition was more pronounced in animals that had received the combination therapy (Fig. 1D) with a significant synergy index (*S* = 3,476 ± 2,390). The robustness of the antiviral effect of the combined therapy was confirmed by immunohistochemical analysis of HBcAg expression (Fig. 2A), as well as by a quantitative method, determining the number of core-positive nuclei per field (Fig. 2B) and the area of HBcAg cytoplasmic staining per field (Fig. 2C). IFN-α or IL-15 alone reduced the presence of core protein in the cytoplasm of hepatocytes but had little effect on the expression of core protein in the nuclei. In contrast, when combined, IFN-α and IL-15 were able to eliminate nuclear core protein expression, showing strong synergy (*S* = 4,673 ± 1,853).

The direct antiviral effects of the different treatments were also analyzed by measuring the viral load in serum and hepatic HBV DNA replicative intermediates by Southern blotting. In all treatment groups, viral loads in the serum were significantly reduced and HBV DNA replicate intermediates in the liver were undetectable (Fig. 2D and E).

As an important aspect of the immune response against HBV, the presence of antibodies against HBsAg was measured by ELISA 20 days after treatment. Animals that had received IFN-α alone or the combination of IFN-α and IL-15 developed anti-HBsAg, and antibody levels were higher in the group receiving the combination treatment (data not shown).

### The IL-15–IFN-α combination treatment induces immune-mediated liver damage in HBVTg mice.

**FIG 3:**
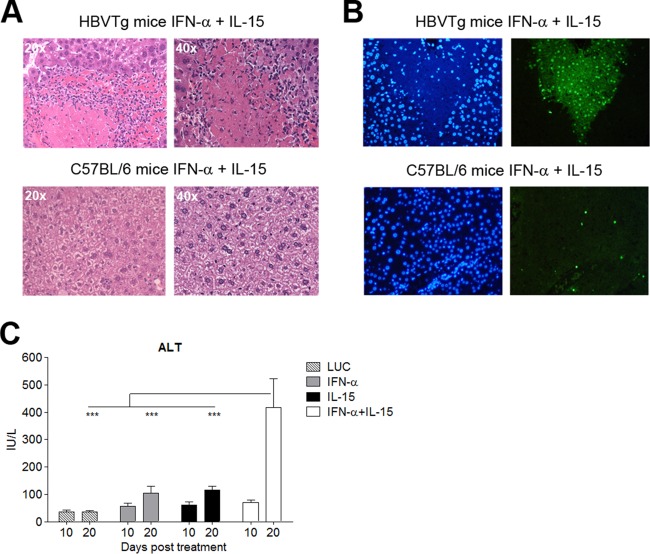
The IL-15–IFN-α combination treatment induces immune-mediated liver damage in HBVTg mice but not in WT mice. (A) Representative images of necroinflammatory lesions in HBVTg (top) and C57BL/6 (bottom) mice on day 20 after vector administration. (B) TUNEL-positive nuclei (right) and 4′,6-diamidino-2-phenylindole (DAPI) staining (left) of the same mice. (C) Serum ALT levels in HBVTg mice at days 10 and 20 after vector administration. Results are expressed as means ± SEM for 5 to 6 animals/group (***, *P* < 0.001).

Liver histology showed no signs of inflammation at day 10 posttreatment in HBVTg mice treated with IFN-α or the control, while marked portal and periportal infiltrates were observed in the IL-15 and IL-15–IFN-α groups (data not shown), and this pattern remained unchanged on day 20 (data not shown). However, on day 20, the livers of the combination group presented with extensive areas of hepatocellular necrosis surrounded by dense lymphocytic infiltrates (Fig. 3A). TUNEL staining showed large areas of positive nuclei in HBVTg mice treated with IL-15 plus IFN-α, while staining was negative for all other groups (Fig. 3B and data not shown). In parallel, marked hypertransaminasemia was observed in these animals (Fig. 3C). Importantly, neither periportal infiltrates nor liver damage was observed in WT mice subjected to the combination therapy (Fig. 3A and B; also data not shown).

### AAV–IL-15 enhances HBV-specific T cell responses in HBVTg mice.

**FIG 4:**
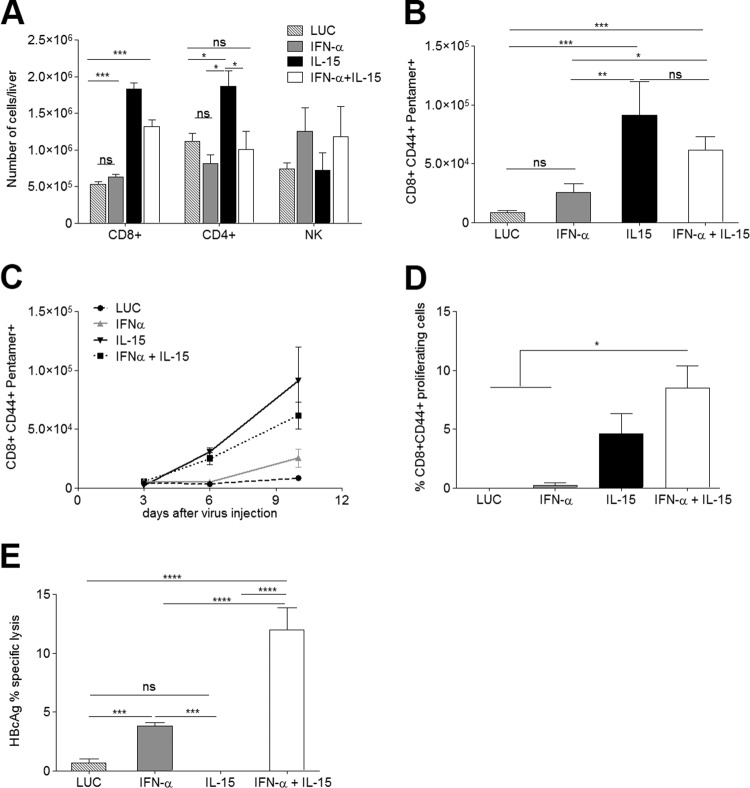
IL-15-induced expansion of HBcAg-specific CD8^+^ T cells requires combination with IFN-α to render them functional CTLs. The mononuclear liver infiltrate was isolated and analyzed on day 10 after vector administration. (A and B) The absolute numbers of CD8^+^, CD4^+^, and NK cells per liver (A) and the number of HBcAg-specific CD8^+^ T cells per liver (B) are shown (*n* = 7). (C) The increase in the number of HBcAg-specific CD8^+^ T cells was analyzed over time (*n* = 3/time point). (D) Proliferative response of HBcAg-specific CD8^+^ T cells upon peptide stimulation. *In vitro* stimulation was performed in triplicate with cells obtained from 3 mice per group. (E) Proportion of lysed HBcAg-loaded target cells *in vivo* (*n* = 5). Results are representative of 3 independent experiments. All graphs show means ± SEM. *, *P* < 0.05; **, *P* < 0.01; ***, *P* < 0.001; ****, *P* < 0.001; ns, nonsignificant.

To explore the nature of the liver infiltrate further, its cellular composition was analyzed in more detail. IL-15 alone or in combination with IFN-α induced an increase in total lymphocyte numbers (data not shown). While IL-15 induced a marked rise in the number of CD8^+^ T cells and a moderate elevation of CD4^+^ T cells (Fig. 4A), only the number of CD8^+^ cells, not that of CD4^+^ T cells, was also significantly increased in the combination treatment group. Another cell type that has been implicated in virus control and clearance (reviewed in reference 27), the NK cell, was found in greatly elevated numbers in response to IFN-α; however, IL-15 seemed to partially counteract this expansion in the combination therapy (Fig. 4A).

Since CD8^+^ T cells are one of the key players in clearing acute HBV infection, the number of HBV-specific CD8^+^ T cells in the liver was determined using H-2Kb pentamers specific for HBcAg epitope C_93–100_. Both IL-15- and IFN-α–IL-15-treated mice showed significantly more intrahepatic CD8^+^ CD44^+^ pentamer-positive T cells than the IFN-α and control groups (Fig. 4B and data not shown). Similar results were obtained by using pentamers specific for HBsAg (data not shown); however, no OVA-specific T cells were detected in the different treatment groups (data not shown). To further characterize the response, the numbers of HBV-specific CD8^+^ lymphocytes in the liver were analyzed at different time points after treatment. While in IFN-α-treated mice HBcAg-specific CD8^+^ T cells were detected in very low numbers at day 3 after treatment and increased only moderately thereafter, the number of pentamer-positive cells in the livers of animals treated with IL-15 or the combination increased significantly over time. The increase was highest in the IL-15 treatment group (Fig. 4C). Only a very low pentamer-positive population could be detected in control animals, and this population is likely to represent the tolerant HBcAg-specific population. The response to HBsAg reflected the response to HBcAg in all groups (data not shown). Furthermore, while CD8^+^ lymphocyte expansion was observed in the livers of C57BL/6 mice treated with IL-15 or IL-15–IFN-α, no HBV- or OVA-specific CD8 T cells were detected (data not shown). When isolated intrahepatic lymphocytes (IHL) were stimulated *ex vivo* on day 10, a robust proliferative response to C_93–100_ (MGLKFRQL) could be observed in IL-15- and IL-15–IFN-α-treated animals, and this response was more intense with the combination treatment (Fig. 4D).

To test if HBV-specific CD8 T cells were able to exert effector functions, an *in vivo* killing assay (IVK) was performed 10 days after the administration of the different treatments. Splenocytes isolated from HBVTg or WT mice were loaded with epitope C_93–100_ or with an irrelevant peptide (OVA_257–264_), labeled with 5 μM or 0.5 μM CFSE, respectively, and transferred i.v. to the groups of mice treated as before. Killing was analyzed 24 h after transfer. No specific lysis was detected in animals treated with the control or IL-15 alone, while in mice that had received IFN-α or IFN-α plus IL-15, specific killing of HBV peptide-loaded cells could be observed (Fig. 4E). The percentage of lysis was significantly higher with the combination treatment than with IFN-α alone. Similar results were obtained in an IVK assay using HBsAg epitope S_208–215_ (ILSPFLPL) (data not shown). No HBV-specific CD8^+^ T cells or cytotoxic activity was seen in WT mice (data not shown).

### CD8^+^ T cells play an essential role in the antiviral response to HBV.

**FIG 5:**
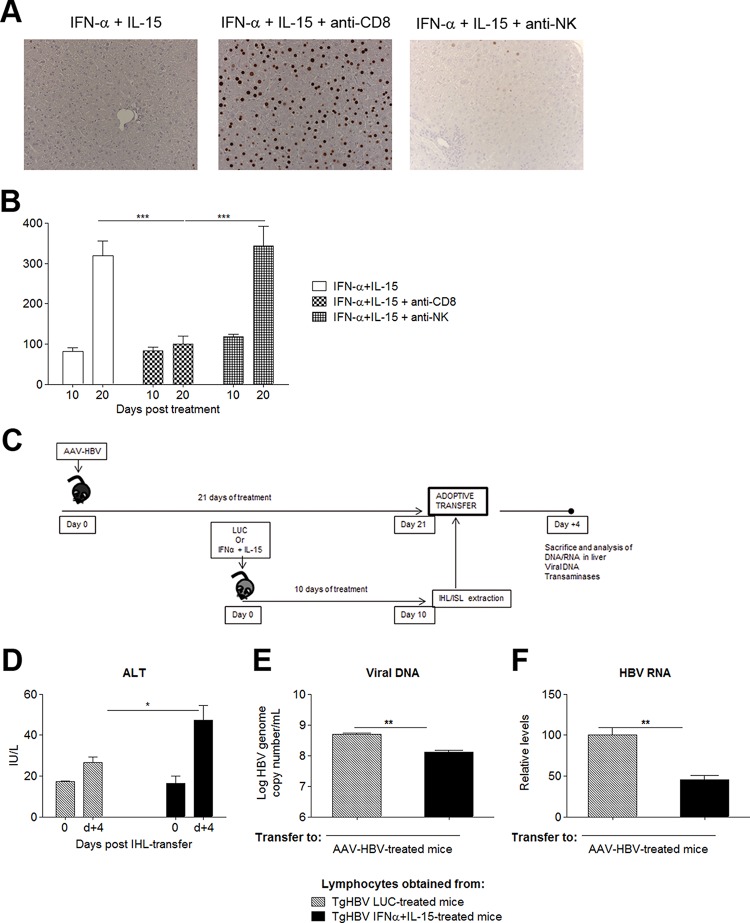
Combined IL-15–IFN-α treatment confers partial protective CD8 immunity. (A) HBVTg mice treated with AAV–IL-15 plus AAV–IFN-α were depleted of CD8^+^ or NK cells. Hepatic HBcAg expression was analyzed 10 days later. Representative images from each group are shown. (B) Serum ALT levels (IU/liter) in HBVTg mice depleted of CD8^+^ or NK cells at day 10 after vector administration. (C) Scheme of the adoptive transfer protocol. (D) Serum ALT levels at day 0 and day 4 (d+4) post-IHL transfer. (E and F) HBV genome copy numbers (E) and HBV RNA copies (F) on day 4 after IHL transfer. Results are normalized to GADPH levels. All data are presented as mean (*n*, 3 to 4) ± SEM. *, *P* < 0.05; **, *P* < 0.01; ***, *P* < 0.001.

To investigate the importance of CD8^+^ T cells and NK cells in mediating the antiviral response elicited by the combination therapy, HBVTg mice treated with IL-15 plus IFN-α were divided into three groups. Group 1 was depleted of CD8^+^ T cells; group 2 was depleted of NK cells; and group 3 received an isotype control antibody. The expression of HBcAg in the liver, which appeared to be a sensitive indicator of the intensity of the antiviral response, was assessed by IHC. As shown in Fig. 5A, depletion of CD8^+^ lymphocytes completely abrogated the antiviral effect of the combination therapy, while NK cell depletion had little impact on the antiviral response. Moreover, while the combined treatment resulted in an elevation of serum transaminases, this alteration was absent in the treated transgenic mice subjected to depletion of CD8^+^ T cells (Fig. 5B). These findings indicate that CD8^+^ lymphocytes are the key players in the antiviral response and that the disappearance of virus from the liver is not solely caused by a direct antiviral effect of the cytokines.

Importantly, transfer of splenic and intrahepatic lymphocytes from HBVTg mice treated with IL-15 plus IFN-α, but not from those receiving the LUC control, to C57BL/6 mice injected with an AAV vector carrying the HBV 1.3 genome—another model of chronic HBV hepatitis (28)—induced moderate hypertransaminasemia as well as a reduction in HBV viremia (Fig. 5C to E). Additionally, significantly lower levels of hepatic HBV RNA could be observed at day 4 after transfer (Fig. 5F), while DNA levels remained unchanged (data not shown). In contrast, LUC DNA and RNA expression did not change in control mice infected with AAV-LUC prior to lymphocyte transfer (data not shown). Taken together, these data suggest that the antiviral effector functions of lymphocytes rescued from tolerance by IL-15–IFN-α treatment are not a bystander effect and can be transferred to chronically HBV infected mice.

### IL-15 upregulates PD-1 and PD-L1 in the inflammatory infiltrate of the liver, an effect counteracted by IFN-α.

**FIG 6:**
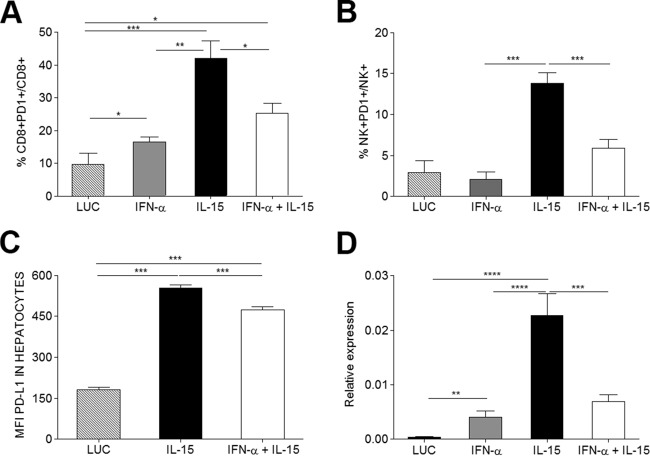
IL-15-induced expression of PD-1 and PD-L1 on liver infiltrates is reduced by IFN-α. (A and B) Ten days after vector administration, PD-1 expression on intrahepatic CD8^+^ (A) and NK^+^ (B) cells was analyzed by flow cytometry. (C) Mean fluorescence intensity (MFI) of PD-L1 expressed on the surfaces of purified hepatocytes. (D) Relative expression of PD-1 in RNA extracted from liver. Results are expressed as means (*n* = 6) ± SEM. *, *P* < 0.05; **, *P* < 0.01; ***, *P* < 0.001; ****, *P* < 0.0001.

PD-1 and PD-L1 constitute a key control point of the CD8^+^ T cell response and have been thought to negatively regulate viral responses (5). Since both PD-1 and PD-L1, the receptor and its ligand, can be upregulated by IFN-α and IL-15 (26, 29–31), their expression was analyzed on hepatocytes and IHL. The proportion of intrahepatic PD-1^+^ CD8^+^ cells was increased in all treatment groups, especially in the presence of IL-15 (Fig. 6A). Surprisingly coinfection with IFN-α seemed to offset this effect to a significant degree. On NK cells, PD-1 upregulation was observed only in the context of IL-15, and again, coadministration of IFN-α seemed to dampen this effect (Fig. 6B). When PD-L1 expression on the surfaces of hepatocytes (Fig. 6C) and relative RNA levels in liver extracts (Fig. 6D) were analyzed, they were also found to be increased in IL-15-treated mice. However, as in lymphocytes and NK cells, combination treatment with IFN-α reduced IL-15-induced PD-L1 expression. For intrahepatic dendritic cells, the same observation was made (data not shown).

### PD-L1 blockade enhances the rescue effect of IL-15.

**FIG 7:**
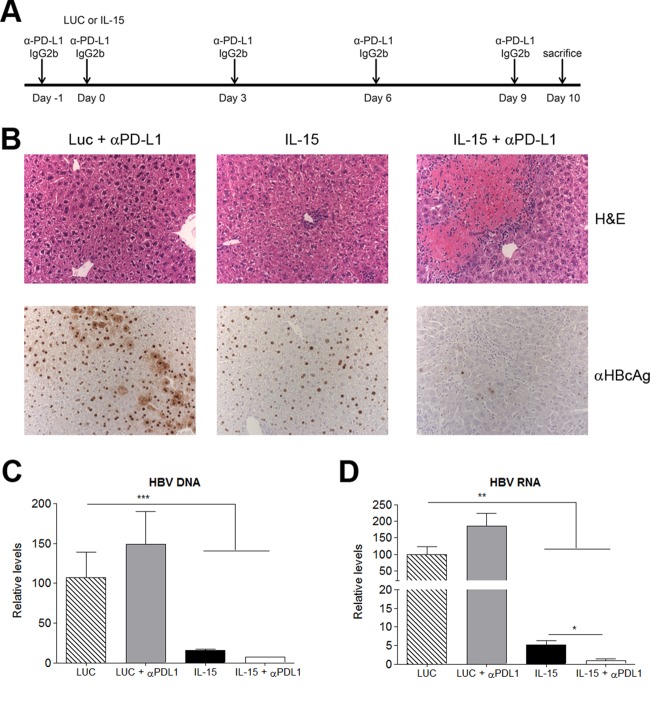
The combination of IL-15 therapy with PD-L1 blockade induces immune-mediated liver damage in HBVTg mice and a strong antiviral effect. (A) Treatment scheme. (B) Representative images of liver sections stained with H&E (upper panels) or stained for core antigen (lower panels). (C and D) HBV (C) and HBV RNA (D) relative levels on day 10 after vector administration. Results are normalized to GADPH levels. Data are presented as means (*n* = 5) ± SEM. *, *P* < 0.05; **, *P* < 0.01; ***, *P* < 0.001.

Since the PD-1–PD-L1 interaction has been implicated in the induction and maintenance of peripheral tolerance (32, 33) and exhaustion (reviewed in reference 34), the effect of PD-L1 expression on the effector functions of IHL expanded in IL-15-treated HBVTg mice was investigated. An anti-PD-L1 blocking antibody was administered intraperitoneally before and after injection of AAV–IL-15 or a control (Fig. 7A). IHC performed on day 10 revealed large areas of necrosis (Fig. 7B, top) and a simultaneous rise in ALT levels (data not shown), as well as an almost-complete disappearance of nuclear HBV core protein from the livers of animals treated with IL-15 in combination with anti-PD-L1 (Fig. 7B, bottom). Analysis of HBV DNA and RNA levels in the liver by quantitative PCR showed that IL-15 alone or in combination with anti-PD-L1 significantly reduced both HBV DNA (Fig. 7C) and RNA (Fig. 7D) levels; the decrease in HBV RNA levels was significantly more pronounced in the last group. Importantly, IL-15 plus PD-L1 blockade did not cause any tissue damage in wild-type mice. Unexpectedly, PD-L1 blockade in mice that had received IFN-α–IL-15 treatment did not augment the infiltration of CD8 cells or their response over that with combination treatment alone. Only a very subtle effect, if any, on the reduction in the number of HBcAg^+^ cells could be observed (data not shown).

### Treatment with IL-15 plus IFN-α can restore effector function and expand the HBV-specific CD8 population in some CHB patients.

**FIG 8:**
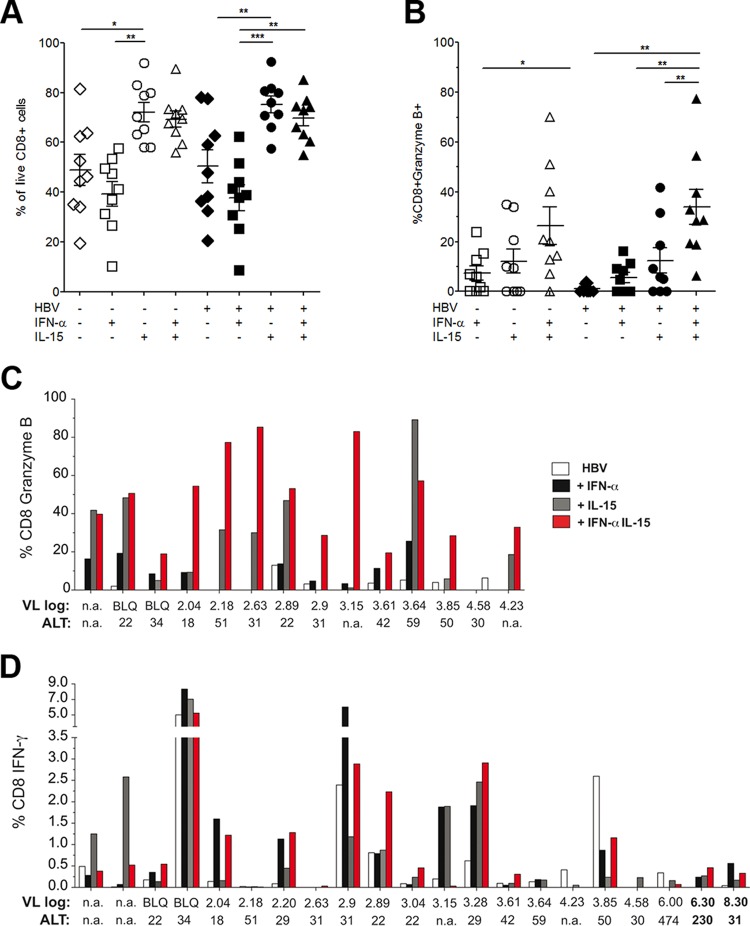
Combination treatment with IL-15 and IFN-α can restore partial CD8 effector function in some patients with chronic hepatitis B. PBMCs from patients with CHB were cultured in IL-2-supplemented medium in the presence/absence of HBV peptide and the indicated cytokines. (A) Cumulative data showing the percentage of CD8 T cells after 10 days of culture. Numbers of live CD8 T cells were measured by using a live/dead staining kit to exclude dead cells. (B) Cumulative data showing the percentage of granzyme B-positive CD8^+^ cells in the presence of IFN-α, IL-15, or both in the absence or presence of HBV peptide stimulation. (C and D) Individual responses of CHB patients, expressed as the percentage of CD8^+^ cells producing granzyme B (C) or IFN-γ (D). Data represent means ± SEM. *, *P* < 0.05; **, *P* < 0.01; ***, *P* < 0.001. Viral loads (VL), expressed as log_10_ IU per milliliter, and ALT levels, expressed as IU per liter, are given below the graphs. n.a., not available; BLQ, below the limit of quantification.

To test if our findings would be transferable to chronically infected patients, a first *in vitro* study with PBMC samples from a small cohort of patients with CHB was performed. Upon stimulation in IL-2-supplemented medium in the presence or absence of an HBV peptide and the cytokines indicated in Fig. 8, the addition of IL-15 resulted in unspecific bulk expansion of the live CD8 T cell population in culture, while IFN-α reduced the population (Fig. 8A). Interestingly, as in the mouse study presented in this paper, IL-15 partially compensated for this effect. In the presence of both cytokines, the overall proportion of HBV-specific granzyme B-producing CD8 T cells (Fig. 8B), as well as the number of patients responding (Fig. 8C), was significantly increased relative to that with either cytokine alone, especially in the presence of an HBV peptide. The percentage of IFN-γ-producing CD8 T cells upon HBV peptide stimulation was also increased by either IFN-α or IL-15 alone or by their combination in approximately half of the 21 patients with CHB tested (Fig. 8D). Because of the global CD8 expansion induced by the combination, the absolute increase in HBV-specific CD8 T cells can be expected to be even more significant. Further studies will require HLA/peptide dextramers to dissect antigen-specific and bystander boosting in a larger cohort of patients with CHB.

## DISCUSSION

The hallmark of CHB is immune dysfunction, which results in persistent infection (3, 4). Because of their stimulatory properties, cytokines such as IFN-α and IL-15 are considered good candidates to aid the functional recovery of HBV-specific lymphocytes (17, 35), and recent work has also highlighted the potential of IFN-α to stimulate cell-intrinsic antiviral mechanisms in HBV-infected hepatocytes (9). However, while IFN-α therapy could strongly drive the expansion and activation of NK cells, it did not reactivate HBV-specific T cells in CHB patients (7, 8). To investigate whether it would be possible to increase the antiviral potential of IFN-α and to more efficiently activate the adaptive arm of the immune response by combining it with the stimulatory cytokine IL-15, an HBVTg mouse model was used (20). These mice present with dysfunctional, unresponsive HBV-specific CD8 T cells and have been widely used to test immunostimulatory therapies aimed at clearing HBV infection (22, 23, 36, 37).

The presence of cytoplasmic HBcAg is an indicator of active HBV replication, whereas nuclear HBcAg is highly stable, persisting in the absence of HBV replication (38, 39). In accordance with previous findings (9), significant decreases in hepatic HBV DNA and HBV RNA levels were obtained upon infection with AAV-expressed IL-15 or IFN-α alone, indicating an attenuation of HBV transcriptional activity and an abrogation of viral replication. However, a high proportion of hepatocyte nuclei remained HBcAg positive. In stark contrast, coinfection with both cytokines resulted in the complete disappearance of HBV core protein from hepatocytes, likely reflecting the successful initiation of an anti-HBV immune response capable of controlling viral replication. Hence, the efficacy of the combined therapy appeared to depend on the synergistic activities of the two cytokines. In terms of cellular immunity, in line with existing literature, IL-15 triggered a robust expansion of the HBV-specific CD8 T cell population (17, 35), while IFN-α induced differentiation into effector cytotoxic T lymphocytes (CTL) without major cellular expansion (40). When combined, HBV-specific CD8 T lymphocytes not only infiltrated the liver in greater numbers but were also able to exert enhanced cytotoxic activity, as reflected in increased serum ALT levels as well as liver pathology. Importantly, no immune response against an irrelevant H-2Kb peptide was detected, and no HBV- or OVA-specific CD8 T cell responses were present in the livers of C57BL/6 wild-type mice receiving the same treatment. In contrast, IHL from HBVTg mice that had received IL-15 only, although numerous, were not able to kill target cells *in vivo* or induce hepatocellular necrosis. These data support the notion that liver damage was at least partially mediated by the complementary effects of IFN-α and IL-15 on specific CD8 T cells and was not only a bystander effect. The successful recovery of HBV-specific CD8^+^ effector cells with the combination treatment and their importance for viral clearance were further corroborated upon their systemic elimination by repeated anti-CD8 administration: the previously observed antiviral effect of the cytokine coadministration disappeared, and HBc protein remained detectable in hepatocyte nuclei. Therefore, the viral clearance observed in the liver was not due solely to the direct, antiviral effects of IFN-α and/or IL-15.

Although in recent years, a potential antiviral role in chronic HBV infection has been attributed to NK cells (27, 41), the therapeutic approach presented here does not seem to support this notion. Both alone and in combination with IL-15, IFN-α resulted in elevated NK cell numbers; however, in contrast to the results obtained for CD8^+^ cells, depletion of NK cells did not result in significant recovery of HBcAg^+^ hepatocytes. This finding is in line with the observations of Yin et al. (19), who reported an NK-independent effect of IL-15 on HBV.

The exact role of PD-1–PD-L1 interaction in the induction and maintenance of T cell tolerance, as well as in exhaustion, is still being disputed and appears rather complex. However, it could be shown that PD-1 blockade can restore T cell function either directly, by using blocking antibodies (42, 43), or indirectly, by blockade of IFN-α (26, 31). In contrast to the latter reports, in our hands, the high increase in the level of PD-1 expressed on intrahepatic CD8^+^- and NK cells from HBVTg mice treated with AAV–IL-15 alone was significantly reduced in mice that had received the IL-15–IFN-α combination therapy. Thus, our data point to a role of IFN-α in prohibiting IL-15-induced PD-L1 upregulation, an effect that would be crucial for licensing effector T lymphocytes to attack target cells. It was reported previously that PD-1 does not directly mediate apoptosis but that its expression levels correlate to the susceptibility of the cell to apoptosis (29). In this study, pretreatment with common γ–chain cytokines, IL-15 included, induced PD-1 upregulation but did not interfere with expansion, enhancement of some effector functions, or survival. This treatment did, however, inhibit the exertion of certain effector functions. The work presented here points to the synergy of IL-15 and IFN-α in overcoming these obstacles. How this may happen in detail requires more exploration. One possibility is the upregulation of other stimulating cytokines/receptors on either the T cells themselves or another cell type, such as dendritic cells. Our approach is corroborated by a recently published study that investigated the roles of Bim, PD-1, and Socs-1 in the onset and degree of acute liver damage in a T cell receptor (TCR) transgenic model. The authors concluded that strategies aimed at overcoming the block in effector function would be more efficient at clearing chronic hepatitis than those purely aiming at prolonging survival (44).

The functional block could also be overcome by blocking the PD-1–PD-L1 interaction and resulted in extensive areas of hepatocellular necrosis and complete clearance of viral antigens from the livers of IL-15-treated mice, indicating that this therapy is equally effective in restoring the anti-HBV immune response.

Although the use of vector-delivered cytokines—and IL-15 in particular—comes with many potential problems in a clinical setting, our work demonstrated that the synergistic potential of the appropriate combination of proliferation- and effector-inducing cytokines can result in the recovery of dysfunctional T cell populations. This work provides new insight into how to tune existing IFN-α treatment of CHB and also offers a therapeutic approach for chronic viral infections and neoplastic conditions. As shown, preliminary results from human studies indicate that this approach may be successful, although the addition of IL-15 appears to promote global as well as HBV-specific T cell expansion, as recently described for HIV patients (45). Such bystander T cell responses may increase the risk of hepatic flares, in line with the necroinflammatory responses observed in our mouse model, but could also contribute to a heterologous antiviral response (46). Further in depth studies will have to be done to explore the synergistic effects of IFN-α and other proliferation-promoting common γ-chain cytokines and their potential efficacy for CHB patients.
